# Characteristics of Inpatients with False-Negative SARS-CoV-2 PCR Test Results

**DOI:** 10.1017/ash.2021.108

**Published:** 2021-07-29

**Authors:** Antigone Kraft, Jessica Ridgway, Erica Mackenzie, Aniruddha Hazra, Maggie Collison, Cassandra Oehler, Madan Kumar

## Abstract

**Background:** At our institution, the concern for false-negative nasopharyngeal testing for SARS-CoV-2 at the onset of illness led to a general policy of retesting inpatients at 48 hours. For such patients, 2 negative SARS-CoV-2 PCR test results were required prior to discontinuation of COVID-19 control precautions. To assess the utility of routine repeat testing We analyzed patients presenting to our hospital who initially tested negative for SARS-CoV-2 but were found to be positive on repeated testing. **Methods:** All inpatients with symptoms concerning for COVID-19 were tested via nasopharyngeal sample for SARS-CoV-2 by PCR on admission. Patients with continued symptoms and no alternative diagnosis were retested 48 hours later. Testing was performed using either the Roche cobas SARS-CoV-2 RT-PCR assay or the Cepheid Xpert Xpress SARS-CoV-2 test. Between March 17, 2020, and May 10, 2020, we retrospectively analyzed data from patients with false-negative SARS-CoV-2 PCR test results who were subsequently confirmed positive 48 hours later. We evaluated demographic information, days since symptom onset, symptomatology, chest imaging, vital sign trends, and the overall clinical course of each patient. **Results:** During the study period, 14,683 tests were performed, almost half (n = 7,124) were performed through the ED and in the inpatient setting. Of 2,283 patients who tested positive for SARS-CoV-2, only 19 (0.01%) initially tested negative. Patients with initial false-negative test results presented with symptoms that ranged from fever and dyspnea to fatigue and vomiting. Notably, few patients presented “early” in their disease (median, 6 days; range, 0–10 days). However, patients with initial false-negative PCR test results did seem to have consistent imaging findings, specifically bilateral bibasilar ground glass opacities on chest radiograph or computed tomography scan. **Conclusions:** Among inpatients with COVID-19, we found a very low rate of initial false-negative SARS-CoV-2 PCR test results, which were not consistently related to premature testing. We also identified common radiographic findings among patients with initially false-negative test results, which could be useful in triaging patients who may merit retesting. Based on these data, we revised our existing clearance criteria to allow for single-test removal of COVID-19 precautions. Evaluating subsequent reduction in unnecessary testing is difficult given changing community prevalence, increased census, and increased opening to elective procedures. However, given the significant percentage of ED and inpatient testing, removal of repeated testing has likely resulted in a reduction of several thousand unnecessary COVID-19 tests monthly.

**Funding:** No

**Disclosures:** None

